# Supplementary material for: Evidence for a DNA-relay mechanism in ParABS-mediated chromosome segregation
Source: eLife. 2014 May 23;3:e02758. doi: 10.7554/eLife.02758 (PMC4067530; doi:10.7554/eLife.02758)
Supplement: Supplementary file 1. — Strains used in this study and the methods of strains construction. DOI: http://dx.doi.org/10.7554/eLife.02758.026 [file elife02758s001.docx]

**Supplementary file 1. Strains used in this study and methods of strains construction**

## Table S1. Strains used in this study

| Strain name | **Relevant genotype or features** | **Reference** | **Construction method** |
| --- | --- | --- | --- |
| *C. crescentus* strains | | |  |
| CB15N | Synchronizable variant of wild-type CB15*,* also named NA1000 | ([Evinger and Agabian, 1979](#_ENREF_2)) |  |
| CJW3010 | CB15N *parA*::*parA-yfp* | ([Schofield et al., 2010](#_ENREF_4)) |  |
| CJW2966 | CB15N *Cori*::Cori(tetO)pGENT *xyl*::pHPV472 (pXyl-lacI-CFP, tetR-yfp) *creS*::pHL23creS-lacO120tm | ([Montero Llopis et al., 2010](#_ENREF_3)) |  |
| CJW3367 | CB15N *parA*::*parA-yfp* *xyl*::pXCFPN-5-parB | ([Schofield et al., 2010](#_ENREF_4)) |  |
| CJW4762 | CB15N *xyl*::pXGFPN-2-parB | This study | Transformation of CB15N with pXGFPN-2-parB. |
| CJW4902 | CB15N *creS*::pHL32-3’creS-dendra2 | This study | Transformation of CB15N with pHL32-3’creS-dendra2. |
| CJW4914 | CB15N *parA*::pNPTS138-parAup-dendra2-parBdown | This study | Transformation of CB15N with pNPTS138-parAup-dendra2-parBdown. |
| CJW4915 | CB15N *parA*::*parA-dendra2* | This study | Subjected CJW4914 to second crossover and selection for sucrose resistance. |
| CJW4978 | CB15N *xyl*::pXmEos3.2-2-ParB | This study | Transformation of CB15N with pXmEos3.2-2-ParB. |
| CJW5154 | CB15N *parA*::*parA-dendra2 xyl*::pXdendra2C-2-parA | This study | Transformation of CJW4915 with pXdendra2C-2-parA. |
| CJW5156 | CB15N *rplA*::pL1-dendra2 | This study | Transformation of CB15N with pL1-dendra2. |
| CJW5466 | CB15N *Cori*::Cori(tetO)pGENT *xyl*::pHPV472(pXyl-lacI-CFP, tetR-yfp) *1599540*::pHPV560 | ([Viollier et al., 2004](#_ENREF_7)) |  |
| CJW5468 | CB15N *Cori*::Cori(tetO)pGENT *xyl*::pHPV472(pXyl-lacI-CFP, tetR-yfp) *2481399*::pHPV560 | ([Viollier et al., 2004](#_ENREF_7)) |  |
| MT174 | CB15N *parB*::e*gfp-parB* | ([Thanbichler and Shapiro, 2006](#_ENREF_6)) |  |
| *E. coli* strains | | |  |
| BL21(DE3) | Protein expression strain | Novagen |  |
| DH5α | Cloning strain | Invitrogen |  |
| CJW3012 | DH5α/ pNPTS138parAUPeYFPdown | ([Schofield et al., 2010](#_ENREF_4)) |  |
| CJW3244 | DH5α/ pHL32-3’creS-mgfp | ([Cabeen et al., 2010](#_ENREF_1)) |  |
| CJW3326 | DH5α/ pXYFPC-2ParAR195E | ([Schofield et al., 2010](#_ENREF_4)) |  |
| CJW3393 | SM10/ pL1-GFPC-1 | ([Montero Llopis et al., 2010](#_ENREF_3)) |  |
| CJW3890 | BL21(DE3)/ pET24dHT-parA | This study | Transformation of BL21(DE3) with pET24HT-parA. |
| CJW4025 | BL21(DE3)/ pET21b-parB | This study | Transformation of BL21(DE3) with pET21b-parB. |
| CJW4488 | BL21(DE3)/ pET24dHT-parA(R195E) | This study | Transformation of BL21(DE3) with pET24dHT-parA(R195E). |
| CJW4504 | BL21(DE3)/ pET21b-parB(L12A) | This study | Transformation of BL21(DE3) with pET21b-parB(L12A). |
| CJW4763 | DH5α/ pXGFPN-2-parB | This study | Transformation of DH5α with pXGFPN-2-parB. |
| CJW4899 | DH5α/ pXdendra2C-2-parA | This study | Transformation of DH5α with pXdendra2C-2-parA. |
| CJW4900 | DH5α/ pHL32-3’creS-dendra2 | This study | Transformation of DH5α with pHL32-3’creS-dendra2. |
| CJW4913 | DH5α/ pNPTS138-parAup-dendra2-parBdown | This study | Transformation of DH5α with pNPTS138-parAup-dendra2-parBdown. |
| CJW5202 | DH5α/ pL1-dendra2 | This study | Transformation of DH5α with pL1-dendra2. |
| MTLS4272 | TOP10/ pXGFPN-2 (pMT582) | ([Thanbichler et al., 2007](#_ENREF_5)) |  |
| MTLS4261 | TOP10/ pXCFPC-2 (pMT587) | ([Thanbichler et al., 2007](#_ENREF_5)) |  |

**References**

CABEEN, M. T., MUROLO, M. A., BRIEGEL, A., BUI, N. K., VOLLMER, W., AUSMEES, N., JENSEN, G. J. & JACOBS-WAGNER, C. 2010. Mutations in the Lipopolysaccharide biosynthesis pathway interfere with crescentin-mediated cell curvature in Caulobacter crescentus. *J Bacteriol,* 192**,** 3368-78.

EVINGER, M. & AGABIAN, N. 1979. Caulobacter crescentus nucleoid: analysis of sedimentation behavior and protein composition during the cell cycle. *Proc Natl Acad Sci U S A,* 76**,** 175-8.

MONTERO LLOPIS, P., JACKSON, A. F., SLIUSARENKO, O., SUROVTSEV, I., HEINRITZ, J., EMONET, T. & JACOBS-WAGNER, C. 2010. Spatial organization of the flow of genetic information in bacteria. *Nature,* 466**,** 77-81.

SCHOFIELD, W. B., LIM, H. C. & JACOBS-WAGNER, C. 2010. Cell cycle coordination and regulation of bacterial chromosome segregation dynamics by polarly localized proteins. *EMBO J,* 29**,** 3068-81.

THANBICHLER, M., INIESTA, A. A. & SHAPIRO, L. 2007. A comprehensive set of plasmids for vanillate- and xylose-inducible gene expression in Caulobacter crescentus. *Nucleic Acids Res,* 35**,** e137.

THANBICHLER, M. & SHAPIRO, L. 2006. MipZ, a spatial regulator coordinating chromosome segregation with cell division in Caulobacter. *Cell,* 126**,** 147-62.

VIOLLIER, P. H., THANBICHLER, M., MCGRATH, P. T., WEST, L., MEEWAN, M., MCADAMS, H. H. & SHAPIRO, L. 2004. Rapid and sequential movement of individual chromosomal loci to specific subcellular locations during bacterial DNA replication. *Proc Natl Acad Sci U S A,* 101**,** 9257-62.
