# Supplementary material for: Evidence for a DNA-relay mechanism in ParABS-mediated chromosome segregation
Source: eLife. 2014 May 23;3:e02758. doi: 10.7554/eLife.02758 (PMC4067530; doi:10.7554/eLife.02758)
Supplement: Supplementary file 2. — Oligonucleotide primers used in this study and methods of plasmids construction. DOI: http://dx.doi.org/10.7554/eLife.02758.027 [file elife02758s002.docx]

## Supplementary file 2. Oligonucleotide primers used in this study and methods of plasmids construction

## Table S2. Primers used in this study

| Primer # | **Primer name** | **Sequence** |
| --- | --- | --- |
| 986 | parBF | GGGGGTACCATGTCCGAAGGGCGTCGTGG |
| 987 | parBR | GGGGAATTCTCAGATCCCGCGCGTCAGT |
| 1010 | His-ParB-up | CCCCCCATATGTCCGAAGGGCGTCGTGGTCTGG |
| 1288 | fpA | CCCCGCCATATGTCCGCTAATCCTCTCC |
| 1289 | rpA | CCCCCAAGCTTAGGCGGCCTTGGCCTGG |
| 1497 | Dentr-R | CCCGCTAGCTTACCACACCTGGCTGGGCAGG |
| 1631 | parB-HindIII | CAAAAAAGCTTGATCCCGCGCGTCAGTCGGTTG |
| 1841 | DendF-NcoI | AAAAACCATGGTGAACACCCCGGGAATTAACC |
| 1842 | DendR-NotI | AAAAAGCGGCCGCTTACCACACCTGGCTGGGCAG |
| 1888 | parBdownR-EcoRI | TGGAAGCAGGAGCGAGAATTCC |
| 1889 | parAEnd-EcoRI | GCCGCGAATTCGAACGTTACG |
| 1890 | dend2parBR | TTGTTCTTTGGACTTACCACACCTGGCTGGGCAGG |
| 1891 | dend2parBF | CAGCCAGGTGTGGTAAGTCCAAAGAACAAGAACCGTAGC |
| 2345 | L12A-for | GGTCTGGGTCGAGGCGCTTCGGCCCTGCTGGGC |
| 2346 | L12A-rev | GCCCAGCAGGGCCGAAGCGCCTCGACCCAGACC |
| 2348 | AgeIDendra2F | TATAACCGGTCGGCCACCATGAACACCCCGGGAAT |
| 2349 | NheIDendra2stopR | TATAGCTAGCTTACCACACCTGGCTGG |
| 2385 | mEosRLKPacI | TTTTTGTTAATTAAGGCGCCTGCAGGTCGTCTGGCATTGTCAG |
| 2386 | mEos3.2NdeIF | ATATCATATGAGTGCGATTAAGCCAG |

# Methods of plasmid construction

All plasmids generated in this study were subjected to sequencing to confirm that there was no unwanted mutation in the insert.

**pET24dHT-parA and pET24dHT-parA(R195E)**. The *parA* or *parA*_R195E_ open reading frame (ORF) was amplified with primers 1288 and 1289 using CB15N chromosomal DNA or plasmid pXYFPC-2ParAR195E as a template. The PCR product was then digested with NdeI and HindIII and ligated into similarly digested pET24dHT.

**pET21b-parB.** *parB* ORF was amplified with primers 1010 and 1631 using CB15N chromosomal DNA as a template. The PCR product was then digested with NdeI and HindIII and ligated into similarly digested pET21b (Novagen).

**pET21b-parB(L12A).** pET21b-parB(L12A) was generated using the QuickChange site-directed mutagenesis protocol using primers 2345 and 2346 and pET21b-parB as a template.

**pXGFPN-2-parB.** *parB* ORF was amplified with primers 986 and 987 using *C. crescentus* chromosomal DNA as a template, digested with EcoRI and KpnI and ligated into similarly digested pXGFPN-2 (isolated from MTLS4272).

**pNPTS138-parAup-dendra2-parBdown.** pNPTS138parAUPeYFPdown from CJW3012 was cut with EcoRI to remove the linker-eyfp-interparAB-parB-399ntdownstream fragment and ligated with similarly digested linker-dendra2-interparAB-parB-399ntdownstream fragment. In doing so, we swapped out the *eyfp* ORF with *dendra2* ORF. We used sequencing to confirm that the insert was incorporated in the correct orientation. The intergenic sequence between *dendra2* and *parB* in this construct contains no restriction site scar and is identical to that between *parA* and *parB* in the CB15N genome. The linker-dendra2-interparAB-parB-399ntdownstream fragment was generated using joint PCR reactions. Two fragments, linker-dendra2 and interparAB-parB-399ntdownstream, were amplified separately with primers 1889 and 1890 and primers 1888 and 1891, respectively, using an intermediate cloning product provided by Dr. Whitman Schofield as a template. The two fragments, which contain a complementary region at the dendra2-interparAB interface, were mixed and used as a template for amplification using primers 1888 and 1889 to finally generate the linker-dendra2-interparAB-parB-399ntdownstream fragment.

**pXdendra2C-2-parA.** pXCFPC-2 isolated from MTLS4261 was cut with NheI and NdeI to remove the *cfp* ORF. The vector was ligated with similarly digested *parA-dendra2* ORF that was amplified with primers 1288 and 1497 using pNPTS138-parAup-dendra2-parBdown as a template.

**pHL32-3’creS-dendra2.** pHL32-3'creS-mgfp isolated from CJW3244 was digested with NotI and NcoI to remove the *mgfp* ORF and ligated with *dendra2* ORF was amplified with primers 1841 and 1842 using pXdendra2C-2-parA as a template and cleaved with the same restriction enzymes.

**pL1-dendra2.** *dendra2* ORF was amplified with primers 2348 and 2349 using pXdendra2C-2-parA as a template. pL1-GFPC-1 isolated from CJW3393 was cut with AgeI and NheI to remove the *gfp* ORF. The vector was gel extracted and ligated with PCR product cut with the same enzymes.

**pXmEos3.2-2-ParB.** *mEos3.2* ORF was amplified with primers 2385 and 2386 using a template provided by Dr. Pingyong Xu, digested with PacI and NdeI and used to replace the *gfp* ORF in pXGFPN-2-ParB.
