# Supplementary material for: Evidence for a DNA-relay mechanism in ParABS-mediated chromosome segregation
Source: eLife. 2014 May 23;3:e02758. doi: 10.7554/eLife.02758 (PMC4067530; doi:10.7554/eLife.02758)
Supplement: Supplementary file 3. — Custom-built MATLAB function (buildTrace.m) for constructing trajectories of ParB/parS complexes in single cells. DOI: http://dx.doi.org/10.7554/eLife.02758.028 [file elife02758s003.docx]

**Supplementary file 3.** Custom-built MATLAB function (buildTrace.m) for constructing trajectories of ParB/*parS* complexes in single cells.

function ttrace=buildTrace(input, pixelsize)

% buildTrace sorts a maximum of two spots in a cell into two trajectories.

% Disclaimer: This simple program deal specifically with cells that contain

% no more than 2 spots at each time frame. The parameters selected worked

% specifically well with our specific dataset.

% INPUT

% input: frame x 3 matrix: frame, long axis coordinate, short axis

% coordinate from one cell over all frames. No more than 2 spots per cell

% at any given frame.

% pixelsize: pixel size of camera in unit of nm.

% OUTPUT

% ttrace: two trajectories/traces with frame, sd, sl

threshold=500; % this value can be optimized for a particular set of data

inpfr=input(:,1);

inpsl=input(:,2);

inpsd=input(:,3);

ttrace(1).sl=[];ttrace(1).sd=[];ttrace(1).frame=[];

ttrace(2).sl=[];ttrace(2).sd=[];ttrace(2).frame=[];

for frame=1:max(input(:,1))

% identify data points

sl=inpsl(inpfr==frame);

sd=inpsd(inpfr==frame);

if ~isempty(sl) %skip if no incoming spot found

%deal with first frame

if isempty(ttrace(1).sl)

ttrace(1).sl=min(sl);

ttrace(1).sd=min(sd);

ttrace(1).frame=frame;

if length(sl)==2

ttrace(2).sl=max(sl);

ttrace(2).sd=max(sd);

ttrace(2).frame=frame;

elseif length(sl)>2

disp(['Warning: >2 spots in frame:' num2str(frame)])

end

else

%to sort spots into traces

%identify last tracked positions from each trace

sl1=ttrace(1).sl(end);

sd1=ttrace(1).sd(end);

%number of spots already in each trace, for assignment

lngTrace1=length(ttrace(1).sl);

lngTrace2=length(ttrace(2).sl);

if ~isempty(ttrace(2).sl) && ~isempty(ttrace(2).sd)

sl2=ttrace(2).sl(end);

sd2=ttrace(2).sd(end);

end

if length(sl)==1 % incoming spot:1

% determine if trace2 already exists, if not, assign the spot to

% trace1 if distance to the last tracked spot in trace1 is

% less than threshold- nm. Otherwise, compute the distance

% of the spot in the current frame to the two spots in the

% last tracked frame and assign based on minimization of

% the sum of the two distances.

sl11= sl(1); sd11= sd(1);

if isempty(ttrace(2).sl)

sl2=ttrace(1).sl(end);

sd2=ttrace(1).sd(end);

dist1st= sqrt((sl11-sl2)^2+(sd11-sd2)^2);

if dist1st*pixelsize< threshold

ttrace=fillTrace(ttrace,1,lngTrace1,sl11, sd11,frame);

else

ttrace=fillTrace(ttrace,2,lngTrace2,sl11, sd11,frame);

end

else

dist1= (sl11-sl1)^2+(sd11-sd1)^2;

dist2= (sl11-sl2)^2+(sd11-sd2)^2;

if dist2> dist1

ttrace=fillTrace(ttrace,1,lngTrace1,sl11, sd11,frame);

else

ttrace=fillTrace(ttrace,2,lngTrace2,sl11, sd11,frame);

end

end

elseif length(sl)==2 %deal with 2 incoming spots

sl11= sl(1);

sd11= sd(1);

sl22= sl(2);

sd22= sd(2);

% determine if trace2 already exists

if isempty(ttrace(2).sl)

dist1= (sl11-sl1)^2+(sd11-sd1)^2;

dist2= (sl22-sl1)^2+(sd22-sd1)^2;

if dist2> dist1

ttrace=fillTrace(ttrace,2,lngTrace2,sl22, sd22,frame);

ttrace=fillTrace(ttrace,1,lngTrace1,sl11, sd11,frame);

else

ttrace=fillTrace(ttrace,1,lngTrace1,sl22, sd22,frame);

ttrace=fillTrace(ttrace,2,lngTrace2,sl11, sd11,frame);

end

else %score all combinations

dist1= (sl11-sl1)^2+(sd11-sd1)^2;

dist2= (sl22-sl1)^2+(sd22-sd1)^2;

dist3= (sl11-sl2)^2+(sd11-sd2)^2;

dist4= (sl22-sl2)^2+(sd22-sd2)^2;

sit1=dist1+dist4;

sit2=dist2+dist3;

% assign spots to traces

if sit1<sit2

ttrace=fillTrace(ttrace,2,lngTrace2,sl22, sd22,frame);

ttrace=fillTrace(ttrace,1,lngTrace1,sl11, sd11,frame);

else

ttrace=fillTrace(ttrace,1,lngTrace1,sl22, sd22,frame);

ttrace=fillTrace(ttrace,2,lngTrace2,sl11, sd11,frame);

end

end

else

disp(['Warning: >2 spots in frame:' num2str(frame)])

end

end

end

end

end

function result=fillTrace(result,trace, lng, sl, sd, frame)

result(trace).sl(lng+1)=sl;

result(trace).sd(lng+1)=sd;

result(trace).frame(lng+1)=frame;

end
