# Supplementary material for: Evidence for a DNA-relay mechanism in ParABS-mediated chromosome segregation
Source: eLife. 2014 May 23;3:e02758. doi: 10.7554/eLife.02758 (PMC4067530; doi:10.7554/eLife.02758)
Supplement: Supplementary file 4. — Custom-built MATLAB function (findFastSegPhase.m) for identifying the ‘fast’ phase in the segregating trajectory. DOI: http://dx.doi.org/10.7554/eLife.02758.029 [file elife02758s004.docx]

**Supplementary file 4.** Custom-built MATLAB function (findFastSegPhase.m) for identifying the ‘fast’ phase in the segregating trajectory.

function result=findFastSegPhase(frame, coord, pix, fpm)

%findFastSegPhase finds the longest segregation phase that is above an

%arbitrarily selected 'threshold'.

% INPUTS

% frame: a vector with frame number information

% coord: corresponding locus position along the long cell axis in

% unit of pixel

% pix: pixel size of camera size in unit of um

% fpm: frames per minute

% OUPUTS

% result: [start of phase fast (min), end of fast phase (min),

% start of fast phase (um), end of fast phase (um)]

% Last updated: 2014-05-15

threshold=0.008; %identify minimal slope to be considered fast phase.

%arbitrarily selected to best identify fast phase. Apply

%the same number for all.

span=25; %sliding window for smoothing

result=[];

time=frame*(1/fpm);

t1=time(1):0.1:time(end); %interpolate time

coord=coord*pix;

intcoord=interp1(time, coord, t1); %interpolate coordinate

smcoord=smooth(intcoord, span); % smooth coordinate with a window of 'span'

dC=diff(smcoord); %calculate slope at all intervals

%plot interp

figure, plot(t1, intcoord,'g'), hold on

t2=t1(1:end-1);

%% find boundaries

dLC=diff(dC>threshold); % 0, 1 or -1,

t3=t2(1:end-1);

index=dLC(dLC~=0);

t3=t3(dLC~=0);

if ~isempty(index)

%quality check to make sure that first number is 1 and ends with -1;

while index(1)<0

index=index(2:end);

t3=t3(2:end);

end

while index(end)>0

index=index(1:end-1);

t3=t3(1:end-1);

end

%% to identify the longest span of trajectory with slope above threshold

clusterSize=0;

for i=2:2:length(index)

if (t3(i)-t3(i-1))>clusterSize

ind=i;

clusterSize=t3(i)-t3(i-1);

end

end

ind1=find(t1==t3(ind-1));

ind2=find(t1==t3(ind));

startLength=intcoord(ind1); %identify start point

endLength=intcoord(ind2); %identify end point

% to remove spontaneous spikes & to remove traces that don't reach the

% new pole

if (length(intcoord)-ind2)>5 && (max(intcoord)-endLength)<0.5

result=[result;[t3(ind-1) t3(ind) startLength endLength]];

plot(result(1), result(3),'.r',result(2), result(4),'.m'),

else

result=[result; [NaN NaN NaN NaN]];

end

else

result=[result; [NaN NaN NaN NaN]];

end

end
